# *Aspergillus *antigen induces robust Th2 cytokine production, inflammation, airway hyperreactivity and fibrosis in the absence of MCP-1 or CCR2

**DOI:** 10.1186/1465-9921-5-12

**Published:** 2004-09-15

**Authors:** Laura L Koth, Madeleine W Rodriguez, Xin Liu Bernstein, Salina Chan, Xiaozhu Huang, Israel F Charo, Barrett J Rollins, David J Erle

**Affiliations:** 1 Lung Biology Center, Department of Medicine, University of California, San Francisco, California, USA; 2 Cardiovascular Research Institute, University of California, San Francisco, California, USA; 3 Program in Immunology, University of California, San Francisco, California, USA; 4 Gladstone Institute of Cardiovascular Disease, University of California, San Francisco, California, USA; 5 Department of Adult Oncology, Dana-Farber Cancer Institute, Harvard Medical School, Boston, Massachusetts, USA

## Abstract

**Background:**

Asthma is characterized by type 2 T-helper cell (Th2) inflammation, goblet cell hyperplasia, airway hyperreactivity, and airway fibrosis. Monocyte chemoattractant protein-1 (MCP-1 or CCL2) and its receptor, CCR2, have been shown to play important roles in the development of Th2 inflammation. CCR2-deficient mice have been found to have altered inflammatory and physiologic responses in some models of experimental allergic asthma, but the role of CCR2 in contributing to inflammation and airway hyperreactivity appears to vary considerably between models. Furthermore, MCP-1-deficient mice have not previously been studied in models of experimental allergic asthma.

**Methods:**

To test whether MCP-1 and CCR2 are each required for the development of experimental allergic asthma, we applied an *Aspergillus *antigen-induced model of Th2 cytokine-driven allergic asthma associated with airway fibrosis to mice deficient in either MCP-1 or CCR2. Previous studies with live *Aspergillus *conidia instilled into the lung revealed that MCP-1 and CCR2 play a role in anti-fungal responses; in contrast, we used a non-viable *Aspergillus *antigen preparation known to induce a robust eosinophilic inflammatory response.

**Results:**

We found that wild-type C57BL/6 mice developed eosinophilic airway inflammation, goblet cell hyperplasia, airway hyperreactivity, elevations in serum IgE, and airway fibrosis in response to airway challenge with *Aspergillus *antigen. Surprisingly, mice deficient in either MCP-1 or CCR2 had responses to *Aspergillus *antigen similar to those seen in wild-type mice, including production of Th2 cytokines.

**Conclusion:**

We conclude that robust Th2-mediated lung pathology can occur even in the complete absence of MCP-1 or CCR2.

## Background

Monocyte chemoattractant protein-1 (MCP-1, also known as CCL2) and its receptor, CCR2, have been the focus of intense interest due to increasing awareness of their association with debilitating human diseases, including asthma [1-3] and pulmonary fibrosis [4-7]. Since MCP-1 attracts and activates a variety of cells, including monocytes, immature dendritic cells, basophils, natural killer cells, and a subset of T lymphocytes [8-17], MCP-1 may have multiple roles in the immune response. Models of Th1 or Th2 inflammation applied to mice deficient in either MCP-1 or CCR2 have clearly shown important roles for this chemokine and its receptor in the development of inflammation [18-24]. However, results obtained using allergen-induced models of asthma (ovalbumin and cockroach antigen) in CCR2-deficient mice are varied, showing either increased, decreased or unchanged Th2 inflammation and airway hyperreactivity (AHR) [25-27], possibly due to differences in the allergen models or strains of mice used. These experiments with CCR2-deficient mice do not directly address the role of MCP-1, which is just one of several MCP chemokines that can bind to CCR2. Although MCP-1-deficient mice have been reported to have defects in Th2 responses [18,19], the effects of MCP-1 deletion in allergen-induced allergic experimental asthma have not been previously reported.

In addition to Th2 inflammation, airway fibrosis is another important feature of human asthma. Blease and colleagues [28,29] examined the contributions of MCP-1 and CCR2 to the development of fibrosis following intratracheal administration of *Aspergillus fumigatus *conidia to *A. fumigatus *sensitized mice. Airway fibrosis was significantly increased in mice treated with MCP-1 neutralizing antibody and in CCR2-deficient mice. However, these increases in fibrosis were seen in the setting of impaired clearance of conidia and a markedly increased neutrophilic inflammatory response, suggesting that the increased fibrosis might be attributable simply to an impaired antifungal response. Previous studies involving other models of allergic asthma applied to CCR2-deficient mice did not examine whether airway fibrosis occurred in these models or whether development of fibrosis was dependent on CCR2 expression [25-27]. Consequently, the role of MCP-1 and CCR2 in the development of allergen-induced lung fibrosis is not well established.

In this study, we hypothesized that the effects of MCP-1 are mediated through CCR2 and that MCP-1 and CCR2 are independently required for the development of experimental allergic asthma. To test this hypothesis, we subjected mice deficient in either MCP-1 or CCR2 to an *Aspergillus *antigen model of Th2-cytokine-driven allergic asthma associated with significant airway fibrosis and measured pulmonary inflammation, cytokine production, AHR and fibrosis.

## Methods

### Mice

Breeding pairs of *Mcp-1*^+/+ ^and *Mcp-1*^-/- ^mice [19] and *Ccr2*^+/+ ^and *Ccr2*^-/- ^mice [21] were generated as previously described. Mice were bred and maintained under specific pathogen-free conditions in the Laboratory Animal Resource Center at San Francisco General Hospital. All mice were backcrossed nine times with C57BL/6 mice (Jackson Laboratory, Bar Harbor, ME). Deletion of *Mcp-1 *or *Ccr2 *genes was confirmed by PCR. Similar numbers of male and female six-week-old mice were used for the study. The UCSF Institutional Animal Care and Use Committee approved all experimental protocols.

### *Aspergillus *Antigen Sensitization Protocol

The *Aspergillus fumigatus *antigen preparation consisted of a mixture of culture filtrate (300 μg protein/mouse) and mycelial extract (80 μg protein/mouse) in PBS (Cellgro by Mediatech, Inc, Herndon, VA). Culture filtrates and mycelial extract were prepared as described previously [30]. For sensitization, anesthetized six-week old mice were given 50 μl of *Aspergillus *antigen intranasally five times at four-day intervals. Control mice were given 50 μl of PBS according to the same schedule as *Aspergillus *antigen-treated mice. All measurements and samples were obtained from mice four days after the final *Aspergillus *antigen administration, which was 20 days after the first challenge. Our group has previously found that airway reactivity measured four days after the final *Aspergillus *antigen challenge was similar to reactivity measured at earlier time points (on the same day as the final challenge or one day after the final challenge) [30].

### Determination of Airway Reactivity

Mice were anesthetized and paralyzed by intraperitoneal injection of etomidate (28 mg/kg) (Bedford Laboratories, Bedford, OH) and pancuronium bromide (0.1 mg/kg) (Baxter Healthcare Corporation, Irvine, CA). A tracheal cannula was inserted via a midcervical incision and the mice were ventilated using a Harvard model 683 rodent ventilator (9 μl/g tidal volume, 150 breaths per minute) (Harvard Apparatus, Holliston, MA). Using a whole body plethysmograph, airflow resistance was calculated during baseline breathing and in response to serially increasing doses of intravenous acetylcholine chloride (0.032, 0.100, 0.316, 1.00, and 3.16 μg/gm body weight) (Sigma, St. Louis, MO). The log of the concentration of acetylcholine (μg/gm) required for a 200% increase in total lung resistance, designated log PC_200_, was reported.

### Bronchoalveolar Lavage (BAL)

After completion of the airway physiology measurements, the lungs were lavaged five times with 0.8-ml aliquots of sterile PBS. The lavage fluid was pooled and centrifuged, and the cell pellet was treated with red-blood-cell lysing buffer (Sigma, Saint Louis, MO). After being washed, the samples were resuspended in PBS. Total leukocytes were counted using a hemacytometer. Differential cell counts were determined by cytocentrifugation and Diff-Quik staining (Dade Behring Inc., Newark, DE) followed by microscopic examination of at least 300 cells.

### Thoracic Lymph Node Isolation and Lung Histology

Thoracic lymph nodes were harvested from mice exposed to *Aspergillus *antigen. Lungs were then removed en bloc and the left mainstem bronchus was firmly sutured closed. The left lung was removed by cutting the left mainstem distal to the suture. It was then frozen in liquid nitrogen and stored at -70°C until processed for hydroxyproline content. The right lung was inflated to 20 cm water pressure with 10% neutral buffered formalin (VWR Scientific Products, West Chester, PA) and fixed in 10% formalin for more than 48 h. Fixed lungs were embedded in paraffin, sectioned at 5 μm thickness, and stained with either hematoxylin and eosin (H&E), periodic acid Schiff (PAS), or trichrome by the Pathology Department of San Francisco General Hospital using standard protocols. The proportion of peribronchial inflammatory cells that were eosinophils was determined by counting inflammatory cells surrounding airways with lumens of 100–200 μm (measured on the short axis) on H&E stained sections. We analyzed 500 total cells (100 cells from each of five airways) for each animal studied.

### Analysis of Cytokine Production by Cells

To prepare single-cell suspensions for cytokine analyses, isolated lymph nodes were gently minced using a syringe plunger and cells were passed through 70-μm cell strainers. Red blood cells were removed by hypotonic lysis at room temperature. Lymph node cells were counted, centrifuged, and resuspended in RPMI medium 1640 (Cellgro by Mediatech, Inc, Herndon, VA) supplemented with FCS (10% vol/vol) (Hyclone, Logan, UT), penicillin (100 U/ml) (Cellgro by Mediatech, Inc, Herndon, VA), streptomycin (100 μg/ml) (Cellgro by Mediatech, Inc, Herndon, VA), phorbol 12 myristate 13-acetate (PMA) (25 ng/m) (Sigma, Saint Louis, MO), and ionomycin (1 μg/ml) (Sigma, Saint Louis, MO) to a final concentration of 5 million cells per ml. Cells were then aliquoted into 96-well plates and incubated at 37°C. After 40 h, cell supernatants were harvested and stored at -70°C until they were analyzed. ELISA for IL-4, -5, -13 and IFN-γ was performed on stimulated lymph node cell supernatant per the manufacturer's protocols (R&D Systems, Minneapolis, MN).

### Determination of MCP-1

For quantitation of MCP-1 levels in BAL fluid, C57BL/6 wild-type mice were treated with the previously described *Aspergillus *antigen protocol. Four days after the final *Aspergillus *antigen administration, lungs from *Aspergillus *antigen- and PBS-treated mice were lavaged two times with 0.6-ml aliquots of sterile PBS. The samples were centrifuged and the supernatants were collected and stored at -70°C until analysis. ELISA for MCP-1 was performed on cell-free BAL fluid per the manufacturer's protocol (R&D Systems, Minneapolis, MN).

### Measurement of Serum Total IgE Concentration

Sera were obtained from blood collected by cardiac puncture from *Aspergillus *antigen- or PBS-treated mice after airway responsiveness measurement. Serum total IgE concentration was determined by a sandwich ELISA using complementary antibody pairs for mouse IgE (clone R35-72 and R35-118) obtained from Pharmingen (Pharmingen, San Diego, CA) according to the manufacturer's instructions. Color development was achieved using streptavidin-conjugated horseradish peroxidase (Pharmingen, San Diego, CA) followed by addition of HRP substrate (ABTS, Sigma, Saint Louis, MO).

### Determination of Lung Hydroxyproline Content

Lungs were analyzed for hydroxyproline content as previously described [31] with slight modification. Lungs were homogenized in distilled water and incubated with 50% trichloroacetic acid on ice for 20 min. Samples were centrifuged and the pellet was mixed with 12 N hydrochloric acid and baked at 110°C for 14–18 h until samples were charred and dry. The samples were resuspended in 2 ml deionized water by incubating for 72 h at room temperature applying intermittent vortexing. Serial dilutions of trans-4-hydroxy-L-proline standard (Sigma, Saint Louis, MO) were prepared. 200 μl of vortexed sample (or standard) was added to 500 μl 1.4% chloramine T/0.5 M sodium acetate/10% isopropanol (Fisher Scientific, Pittsburgh, PA) and incubated for 20 min at room temperature. Next, 500 μl of Ehrlich's solution (1.0 M p-dimethylaminobenzaldehyde, 70% isopropanol/30% perchloric acid) (Fisher Scientific, Pittsburgh, PA) was added, mixed, and incubated at 65°C for 15 min. After samples returned to room temperature, the optical density of each sample and standard was measured at 550 nm and the concentration of lung hydroxyproline was calculated from the hydroxyproline standard curve.

### Statistical Analysis

Statistical significance for treatment effect was determined by analysis of variance with post-ANOVA t tests corrected for multiple comparisons using Bonferroni adjustment. These statistical analyses were performed using statistical software STATA 5.0 (Stata Corporation, College Station, TX) and R [32] (The R Foundation for Statistical Computing, Vienna, Austria). All tests were two-tailed with a p-value of 0.05 for statistical significance.

## Results

### *Aspergillus *antigen airway challenge induces MCP-1 production

We used a model system that involved repeated intranasal challenges with *Aspergillus *antigen over a 20-day period. To determine whether antigen challenge induces MCP-1 production in the airway, we measured MCP-1 protein levels in BAL fluid from wild-type C57BL/6 mice on day 20. MCP-1 levels were markedly higher in *Aspergillus *antigen-treated mice (46.3 ± 12.7 pg/ml, mean ± SE) than in PBS-treated mice (5.8 ± 1.3 pg/ml), (P = 0.01).

### MCP-1- and CCR2-deficient mice develop airway inflammation in response to *Aspergillus *antigen

**Figure 1:**
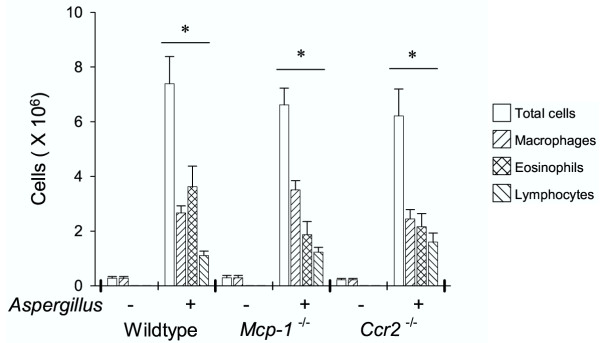
***Aspergillus *antigen induced similar increases in BAL fluid cell counts in wild-type, *Mcp-1*^-/- ^and *Ccr2*^-/- ^mice. **Total cells, macrophages, eosinophils, and lymphocytes are expressed as mean BAL fluid total cell counts ± SE from wild-type, *Mcp-1*^-/- ^and *Ccr2*^-/- ^mice (PBS-treated, N = 5 mice/group; *Aspergillus *antigen-treated, N = 8 mice/group; *Aspergillus *antigen exposure and sample collection are described in methods). Neutrophils represented <0.5% of total cells for all groups. The data shown are from one experiment and representative of three separate experiments. Asterisks (*) indicate values that are statistically significantly different (p < 0.001) compared to PBS controls.

**Figure 2:**
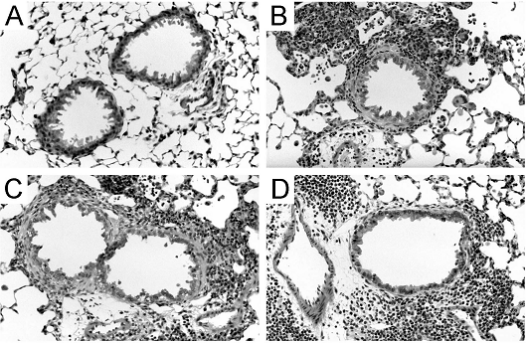
***Aspergillus *antigen-induced lung inflammation appears similar in wild-type, *Mcp-1*^-/- ^and *Ccr2*^-/- ^mice. **H&E stained lung sections from PBS- or *Aspergillus *antigen-treated wild-type, *Mcp-1*^-/- ^and *Ccr2*^-/- ^mice. Representative normal airway from wild-type control mice (A) (similar findings from *Mcp-1*^-/- ^and *Ccr2*^-/- ^control mice are not shown). Representative lung sections from *Aspergillus *antigen-treated wild-type (B), *Mcp-1*^-/- ^(C) and *Ccr2*^-/- ^mice (D) demonstrate intense peribronchiolar and perivascular inflammation. *Aspergillus *antigen exposure and sample collection are described in methods. Magnification: 20× objective.

The *Aspergillus *antigen induction of MCP-1 was accompanied by a significant degree of lung inflammation as assessed by BAL fluid cell counts and lung histology. In wild-type mice, *Aspergillus *antigen induced a >20-fold increase in BAL fluid cell numbers (Fig. 1) and the development of prominent infiltrates in peribronchovascular spaces and scattered infiltrates in the lung parenchyma (Fig. 2A and 2B). The inflammatory infiltrates consisted of numerous eosinophils as well as other cell types.

To determine the airway inflammatory response to *Aspergillus *antigen in the absence of MCP-1 or its receptor, CCR2, we used mice with targeted disruptions of the genes that encode MCP-1 and CCR2. Since mouse strain differences are associated with major differences in antigen reactivity in many model systems, the mice used here were produced by extensive backcrossing into a C57BL/6 genetic background. Both MCP-1- and CCR2-deficient mice developed marked airway inflammation in response to *Aspergillus *antigen (Figs. 2C and 2D). The BAL fluid cell counts from *Aspergillus *antigen-treated MCP-1- and CCR2-deficient mice revealed significantly greater numbers of all cell types than in PBS-treated controls (p < 0.001). The numbers of macrophages, lymphocytes and neutrophils were not significantly different from those in *Aspergillus *antigen-treated wild-type mice (Fig. 1). The BAL fluid eosinophil response in MCP-1- and CCR2-deficient mice was slightly (~30–40%) smaller than in wild-type mice, but this difference did not reach statistical significance (Fig. 1). The fraction of peribronchial inflammatory cells that were eosinophils was not significantly different among wild-type mice (51 ± 13%, mean ± standard deviation), CCR2-deficient mice (52 ± 6%), and MCP-1-deficient mice (37 ± 13%) (N = 5 mice/group). These findings indicate that there was a robust inflammatory response to *Aspergillus *antigen even in the absence of MCP-1 or CCR2.

### MCP-1- and CCR2-deficient mice develop AHR and produce mucus in response to *Aspergillus *antigen

**Figure 3:**
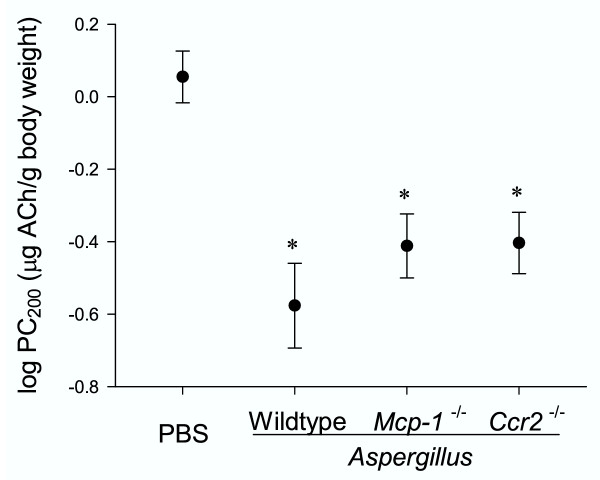
***Aspergillus *antigen induced AHR in wild-type, *Mcp-1*^-/- ^and *Ccr2*^-/- ^mice. **Airway reactivity in response to intravenous acetylcholine was measured invasively. Data are expressed as log PC_200 _and lower values indicate higher airway response. *Aspergillus *antigen exposure and the airway measurement protocol are described in methods (PBS-treated, N = 12 mice; *Aspergillus *antigen-treated, N = 8–10 mice/group;). The data shown are from one experiment and representative of three separate experiments. Asterisks (*) indicate values that are statistically significantly different (p < 0.001) compared to PBS controls.

To determine airway reactivity to acetylcholine in mice exposed to *Aspergillus *antigen or to vehicle (PBS) alone, we compared airway reactivity of PBS- and *Aspergillus*-antigen-treated mice 4 days after the final challenge as described in the methods section. Measurements from this time point were previously found to be comparable to those from earlier time points [30]. In the experiment shown in Fig. 3, the PBS-treated group included a mixture of wild-type, *Mcp-1*^-/-^, and *Ccr2*^-/- ^mice since preliminary experiments showed similar airway reactivity between PBS-treated wild-type, *Mcp-1*^-/-^, and *Ccr2*^-/- ^mice (not shown). *Aspergillus*-antigen-treated wild-type, *Mcp-1*^-/-^, and *Ccr2*^-/- ^mice each had significantly lower PC_200 _values than did PBS-treated controls (P < 0.001), indicating the development of AHR (Fig. 3). Although there appeared to be a trend toward less airway reactivity in *Aspergillus*-antigen-treated *Mcp-1*^-/- ^and *Ccr2*^-/- ^mice than in *Aspergillus*-antigen-treated wild-type mice, this trend was not statistically significant and was not observed in two additional *Aspergillus*-antigen-challenge experiments comparing wild-type mice to either *Mcp-1*^-/- ^or *Ccr2*^-/- ^mice separately (data not shown).

**Figure 4:**
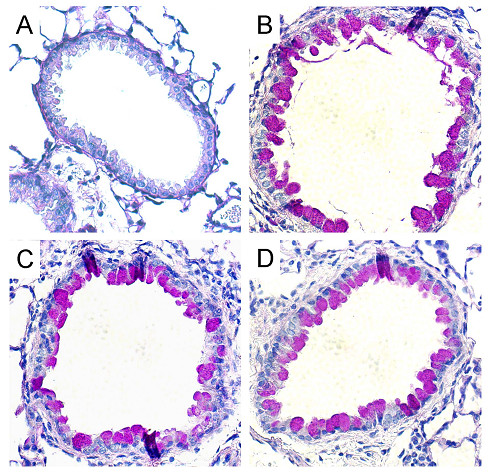
***Aspergillus *antigen induced goblet cell hyperplasia in wild-type, *Mcp-1*^-/- ^and *Ccr2*^-/- ^mice. **Representative PAS-stained lung sections from PBS-treated wild-type mice (A) showed minimal PAS-positive staining (similar findings from *Mcp-1*^-/- ^and *Ccr2*^-/- ^control mice are not shown). *Aspergillus *antigen-treated wild-type (B), *Mcp-1*^-/- ^(C) and *Ccr2*^-/- ^mice (D) showed magenta staining in epithelial cells, which represents mucus. *Aspergillus *antigen exposure and sample collection are described in methods. Magnification, 40× objective.

To determine if *Aspergillus*-antigen challenge results in increased mucus production, we analyzed lung histology by PAS-staining. As shown in Fig. 4A, there was minimal PAS staining in the airway epithelium of control mice. In contrast, *Aspergillus*-antigen-treated mice from all three groups showed accumulation of PAS-stained material in epithelial cells (Fig. 4B,4C,4D), indicating that *Aspergillus *antigen airway challenge resulted in mucus production by goblet cells. These findings indicate that *Aspergillus *antigen induces AHR and mucus production even in the absence of MCP-1 or CCR2.

### Th2 cytokine and IgE production is similar in *Aspergillus *antigen-treated wild-type, *Mcp-1*^-/- ^and *Ccr2*^-/- ^mice

**Figure 5:**
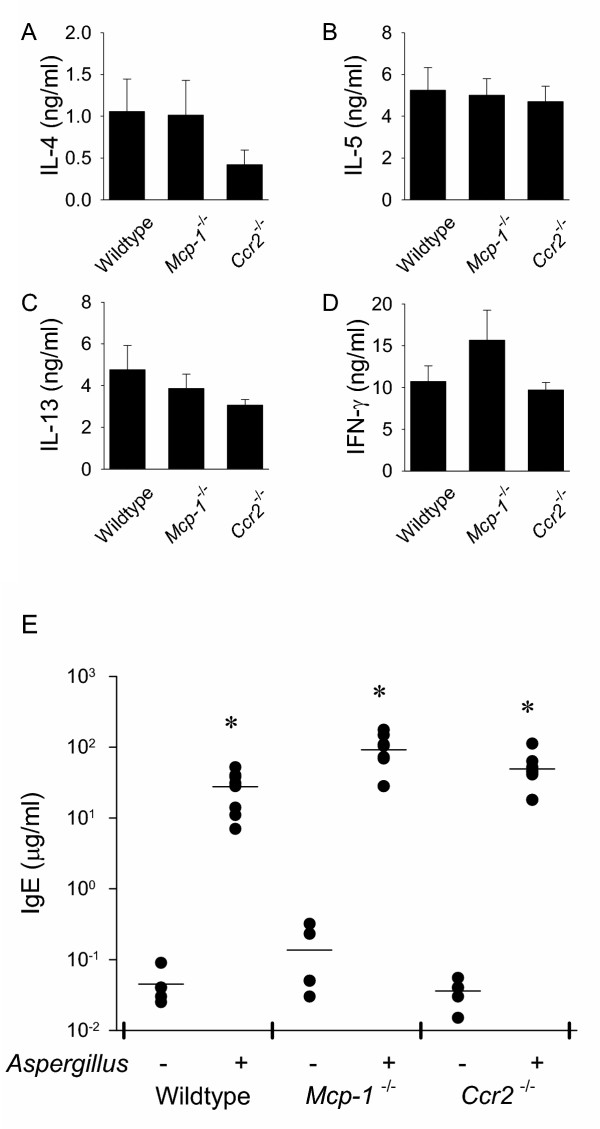
***Aspergillus *antigen-treated wild-type, *Mcp-1*^-/- ^and *Ccr2*^-/- ^mice demonstrated intact Th2 cytokine production and induction of IgE. **For cytokine determination, draining lymph node cells from *Aspergillus *antigen-treated wild-type, *Mcp-1*^-/- ^and *Ccr2*^-/- ^mice were isolated and stimulated with PMA/ionomycin for 40 hr and cytokine levels for IL-4 (A), IL-5 (B), IL-13 (C), and IFN-γ (D) were quantitated by ELISA. Serum IgE (E) from *Aspergillus *antigen-treated wild-type, *Mcp-1*^-/- ^and *Ccr2*^-/- ^mice and control mice were measured by ELISA. In (A-D), bars represent mean ± SE; in (E), results are expressed as the common log of IgE concentration where each circle represents a single PBS- or *Aspergillus *antigen-treated mouse and horizontal lines represent the mean of each group (PBS-treated, N = 5 mice/group; *Aspergillus *antigen-treated, N = 8–9 mice/group). *Aspergillus *antigen exposure and sample collection are described in methods. Asterisks (*) indicate values that are statistically significantly different (p < 0.001) compared to PBS controls.

To determine if deletion of MCP-1 or CCR2 alters the cytokine response to *Aspergillus *antigen, we assayed Th1 and Th2 cytokines in stimulated cell supernatants prepared from thoracic lymph nodes isolated from *Aspergillus *antigen-treated mice. (PBS-treated mice had much smaller thoracic lymph nodes and it was not possible to reliably obtain sufficient numbers of cells from these mice for comparison.) MCP-1- and CCR2-deficient mice had concentrations of the cytokines IL-4, IL-5, IL-13 and IFN-γ generally similar to those in wild-type mice (Fig. 5A,5B,5C,5D). There was a trend toward lower IL-4 production in cells from *Ccr2*^-/- ^mice, but this difference was not statistically significant. In addition, sera from *Aspergillus*-antigen-treated mice and control mice were assayed for serum total IgE levels. As shown in Fig. 5E, *Aspergillus *antigen induced increases in serum IgE in wild-type, *Mcp-1*^-/-^, and *Ccr2*^-/- ^mice similar to those in control mice.

### *Aspergillus *antigen-induced lung fibrosis develops in the absence of MCP-1 or CCR2

**Figure 6:**
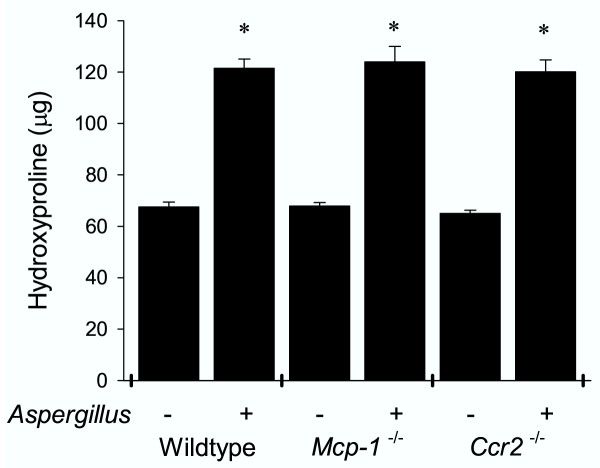
***Aspergillus *antigen induced similar lung fibrosis in wild-type, *Mcp-1*^-/- ^and *Ccr2*^-/- ^mice. **Left lungs from *Aspergillus *antigen- or PBS-treated wild-type, *Mcp-1*^-/- ^and *Ccr2*^-/- ^mice were analyzed for total hydroxyproline content as described in methods. Results are expressed as mean ± SE (N = 10 mice/group). *Aspergillus *antigen exposure and sample collection are described in methods; data are representative of two separate experiments. Asterisks (*) indicate values that are statistically significantly different (p < 0.001) compared to PBS controls.

**Figure 7:**
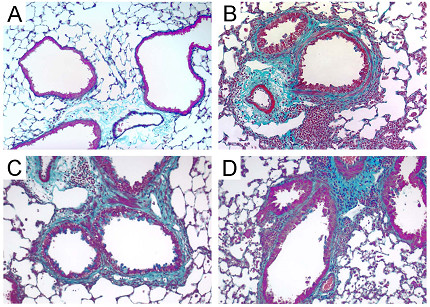
**Increased airway subepithelial collagen deposition after treatment with *Aspergillus *antigen. **Representative lung sections from PBS-treated mice show minimal trichrome staining around small airways (A) (similar findings from *Mcp-1*^-/- ^and *Ccr2*^-/- ^control mice are not shown). Increased trichrome staining is noted around small airways in *Aspergillus *antigen-treated wild-type (B), *Mcp-1*^-/- ^(C) and *Ccr2*^-/- ^(D) mice. Blue staining around airways represents collagen. *Aspergillus *antigen exposure and sample collection are described in methods. Magnification, 20× objective.

To determine whether *Aspergillus *antigen-induced airway fibrosis develops in the absence of MCP-1 or CCR2, we measured lung hydroxyproline content in PBS- and *Aspergillus*-antigen-challenged mice (Fig. 6). *Aspergillus *antigen treatment resulted in a two-fold increase in lung hydroxyproline, a measure of collagen content. This effect was very similar in wild-type, *Mcp-1*^-/-^, and *Ccr2*^-/- ^mice. Histopathologically, lung sections from PBS-treated mice had normal lung architecture and minimal evidence of trichrome staining (Fig. 7A). Lung sections from mice treated with *Aspergillus *antigen had clear increases in trichrome staining in a peribronchiolar distribution (Fig. 7B,7C,7D). There were no apparent differences in trichrome staining in wild-type mice as compared to either MCP-1- or CCR2-deficient mice after allergen challenge.

## Discussion

We hypothesized that MCP-1 and its receptor, CCR2, are independently required for the development of *Aspergillus*-antigen-induced allergic asthma. We found that wild-type C57BL/6 mice challenged with *Aspergillus *antigen developed robust Th2 responses associated with pulmonary inflammation, AHR, mucus production and fibrosis. Surprisingly, neither MCP-1 nor CCR2 was critical for the development of these lung pathologies, since robust responses were also seen in mice with deletions of genes encoding either protein. These results demonstrate that neither MCP-1 nor CCR2 are required for the development of experimental allergic asthma induced by exposure to *Aspergillus *antigen.

Our results stand in contrast to some previous reports showing important roles for MCP-1 or CCR2 in other models of allergic asthma [25,27,33]. Although the precise explanation of these differences is not clear, there are several experimental factors that may contribute. For example, the choice of antigen and the route of sensitization differ between models. We used antigens prepared from *Aspergillus*, an important allergen in some people with asthma, and administered it exclusively to the respiratory tract, presumably a relevant route for sensitization in asthma. Previous studies have used ovalbumin [25,26,33] or cockroach antigen [27] and have used intraperitoneal antigen injections to sensitize prior to antigen challenge. CCR2-deficient mice have been shown to have defects in recruitment of antigen-presenting cells to the peritoneum [21,34,35], suggesting that CCR2 could be important for sensitization when antigen is administered to the peritoneum. Another factor that differs between studies is timing. We studied mice at 4 days after the final allergen challenge, when all aspects of the *Aspergillus *antigen-induced experimental asthma phenotype are present. Campbell *et al. *found that the administration of MCP-1 antibody could inhibit AHR in cockroach antigen sensitized and challenged mice at very early time points (1 and 8 h post challenge) but not later (24 h after challenge) [27]. The effect on AHR at 1 and 8 h was ascribed to MCP-1's ability to activate mast cells, which are important in some asthma models but not in others [36]. Genetic background may also be an important factor, since mouse strains vary widely in their response to airway antigen challenge [37]. Previous experimental asthma studies involving CCR2-deficient mice have used mice of mixed genetic backgrounds [25-27], whereas we used mice that had been backcrossed nine times to C57BL/6 and therefore have a more homogenous genetic background. Some of the specifics of our experimental system may therefore account for the lack of a requirement for MCP-1 and CCR2. However, MacLean *et al. *[26] used an allergic asthma model involving ovalbumin, intraperitoneal sensitization, and mice of mixed genetic backgrounds and found that CCR2-deficient mice had intact responses to allergen challenge. This indicates that the lack of a requirement for CCR2 is not unique to a single asthma model. It also highlights the difficulty in pinpointing the experimental factors that account for the diverse results reported by various investigators.

Of note, neither MCP-1 nor CCR2 was critical for inflammatory cell migration to the lungs after *Aspergillus *antigen challenge. We found that *Aspergillus *antigen-induced monocyte recruitment (as measured by counting BAL fluid macrophages) was intact in both MCP-1- and CCR2-deficient mice. While intact alveolar macrophage recruitment in response to airway instillation of *Saccharopolyspora rectivirgula *has been reported in CCR2-deficient mice [38], other *in vivo *models have demonstrated requirements for MCP-1 and CCR2 in monocyte/macrophage recruitment [19,39-42]. Our finding indicates that other chemoattractants are sufficient for maximal monocyte/macrophage recruitment in this *Aspergillus *antigen model. In support of this observation, a recent microarray-based analysis of gene expression changes in a similar asthma model found that 14 different chemokines (including MCP-1/JE) were induced by *Aspergillus *antigen challenge [43]. However, we did find that MCP-1 and CCR2 may have indirect effects on eosinophil recruitment in response to *Aspergillus *antigen. While there was marked eosinophil recruitment to the lungs in MCP-1- and CCR2-deficient mice, there was a trend toward fewer eosinophils than in wild-type mice. Since MCP-1 is not a chemoattractant for eosinophils (which lack CCR2), this trend suggests that MCP-1 may have indirect effects on eosinophil recruitment in this model. A more dramatic decrease of eosinophil recruitment has been seen following neutralization of MCP-1 in another model, but that effect was associated with other signs of impaired Th2 immunity [33]. Although there may be some role for MCP-1 and CCR2 in eosinophil recruitment, robust inflammatory responses to *Aspergillus *antigen occurred even in the complete absence of either of these molecules.

In contrast to our results indicating a robust Th2 response in MCP-1- and CCR2-deficient mice after *Aspergillus *antigen challenge, diminished Th2 cytokine production has been reported in studies of MCP-1 neutralization or deletion in different models [19,20,33,44,45]. In studies involving CCR2-deficient mice, the results have been more heterogenous, suggesting that CCR2 deletion may increase [25,28], decrease [24], or have no effect on Th2 responses [26]. As mentioned previously, the explanation for these different Th2 responses in CCR2-deficient mice is not clear, and may suggest that complex pathways involving other CCR2 ligands or MCP-1 receptors [46] are operational in different models of inflammation. However, if these pathways exist and were important in the model we used, we would have expected to find that deletion of MCP-1 and CCR2 had different effects. Instead, we observed that MCP-1- and CCR2-deficient mice were similar in all respects, including cytokine production, IgE production, and AHR. Our results support the idea that the role of MCP-1 and CCR2 in the development of allergic responses may be dependent upon the experimental model used.

The role of MCP-1 and CCR2 in the development of allergen-induced airway fibrosis has not been extensively explored. Previous findings of increased pulmonary fibrosis in CCR2-deficient mice compared to wild-type mice after treatment with *Aspergillus *conidia were accompanied by neutrophilic inflammation and the inability of CCR2-deficient mice to clear the organism normally [28,29]. Consequently, the persistence of *Aspergillus *organisms in the airway may have altered the fibrotic response. Other studies involving different experimental systems have suggested that MCP-1 and CCR2 may directly or indirectly contribute to the development of fibrosis. Gharaee-Kermani et al. [47] found that MCP-1 directly induced increased production of collagen by cultured fibroblasts, although the role of CCR2 was not explored in that report. MCP-1 and CCR2 may also indirectly influence fibrosis via their effects on inflammatory cells. Previous studies showed that CCR2-deficient mice developed less pulmonary fibrosis in response to three different stimuli, including intratracheal bleomycin instillation, than did wild-type mice [48,49]; however, those studies did not test the requirement for MCP-1 in the development of fibrosis. In C57BL/6 mice, bleomycin induces a robust inflammatory response that consists of neutrophils and lymphocytes, with a smaller component of eosinophils [50], in contrast to our allergen model. Thus, it is possible that the relative abundance or types of recruited cells in response to a particular airway challenge greatly influence the character or extent of lung fibrosis mediated by MCP-1 or CCR2.. Therefore, based on these previously published results we might have expected MCP-1 and CCR2 to be critical to the development of allergen-mediated fibrosis. However, we found that MCP-1-deficient and CCR2-deficient mice each developed marked fibrosis following *Aspergillus *antigen challenge, similar to wild-type mice. Our result, in contrast to the reported requirement for CCR2 in the development of bleomycin-induced pulmonary fibrosis, suggests that different cell types and mediators may be operational in allergen-induced airway fibrosis than those observed in bleomycin-induced lung fibrosis.

## Conclusions

In conclusion, this study demonstrates that pulmonary inflammation, Th2 immune responses, Th2-mediated airway pathology, and lung fibrosis are remarkably intact despite the complete absence of MCP-1 or CCR2 in an *Aspergillus *antigen-driven model of allergic airway disease. Previous studies have demonstrated roles for MCP-1 and CCR2 in other models of inflammation and fibrosis, including different allergic airway disease models [25,27,33]. Those findings indicate that the role of MCP-1 and CCR2 in allergic responses and in fibrosis depends on the models used, although it is difficult to identify which experimental factors determine whether MCP-1 and CCR2 are required. Both MCP-1 and CCR2 may be good therapeutic targets for some diseases. However, the variable involvement of these potential targets in animal models indicates that it may be extremely challenging to predict which human diseases are most likely to benefit from this approach.

## Abbreviations

AHR, airways hyperreactivity; BALF, bronchoalveolar lavage fluid.

## Authors' contributions

LLK conceived of the experiment, carried out all experiments and prepared the manuscript. MWR assisted in collection and analysis of mouse samples. XLB performed all mouse airway measurements. SC and XH performed antigen challenge and assisted in collection and analysis of mouse samples. IFC and BJR provided the targeted knock-out mice, provided expert advice and interpretation of the study's results. DJE participated in the study's design, coordination and final revisions of the manuscript. All authors read and approved the final manuscript.
